# Psychiatric disorders among young doctors: What impact on medical fitness for work?

**DOI:** 10.1192/j.eurpsy.2026.11302

**Published:** 2026-07-31

**Authors:** W. Ayed, S. Chemingui, Y. Sellaouti, M. Mersni, I. Youssfi, G. Bahri, D. Brahim, M. Bani, H. Nefzi, N. Ladhari

**Affiliations:** 1 Occupational Medecine Department, Charles Nicolle Hospital, Tunis; 2 Psychiatric department, Razi hospital, Mannouba, Tunisia

## Abstract

**Introduction:**

Medical training is a demanding journey, marked by intense workloads, increasing responsibilities and frequent exposure to stressful situations. These conditions can undermine the mental health of young doctors, promoting the emergence or worsening of psychiatric disorders.

**Objectives:**

- To Describe the socio-demographic and clinical characteristics of medical trainees with psychiatric disorders.
- To Assess medico-legal decisions regarding fitness for work.

**Methods:**

Retrospective descriptive study of the files of medical trainees who consulted the occupational medicine department of Charles Nicole Hospital between 1 January 2020 and 30 June 2023 for a medical fitness assessment.

**Results:**

Among the fifty medical trainees who consulted our service for an aptitude assessment, psychiatric disorders accounted for 28% of cases (n=14). The average age was 29±45 years. The majority were female (92%). They mainly belonged to medical (86%) and surgical (14%) specialities. The psychiatric disorders represented were: anxiety-depression disorder (n=9), bipolar disorder (n=3), substance abuse (n=1) and burnout (n=1). Two cases reported a suicide attempt. They were undergoing treatment with antidepressants (n=8), antipsychotics (n=4) and mood stabilisers (n=2). The median time between diagnosis and consultation with the occupational health service was 64 days. The medico-legal decisions regarding fitness for work were: continuation of work with restrictions (50%) and temporary unfitness for work (21%). The restrictions indicated were: exclusion from night work (n=7)

**Conclusions:**

Psychiatric disorders among trainee doctors are a serious issue, both in terms of their frequency and their impact on professional ability. Dealing with them requires a multidisciplinary approach combining medical monitoring, psychological support and adjustments to working conditions.

**Disclosure of Interest:**

None Declared

